# The Synergy of Thermally Reduced Graphene Oxide in Amperometric Urea Biosensor: Application for Medical Technologies

**DOI:** 10.3390/s20164496

**Published:** 2020-08-11

**Authors:** Julija Razumiene, Vidute Gureviciene, Ieva Sakinyte, Laurynas Rimsevicius, Valdas Laurinavicius

**Affiliations:** 1 Institute of Biochemistry, Life Science Center of Vilnius University, LT-10257 Vilnius, Lithuania; vidute.gureviciene@gmc.vu.lt (V.G.); ieva.sakinyte@gmc.vu.lt (I.S.); valdas.laurinavicius@bchi.vu.lt (V.L.); 2 Institute of Clinical Medicine, Vilnius University, LT-08661 Vilnius, Lithuania; laurynas.rimsevicius@santa.lt

**Keywords:** amperometric biosensor, thermally reduced graphene oxide, urea, spent dialysate, urine, non-invasive technology

## Abstract

Thermally reduced graphene oxide (TRGO) is a graphene-based nanomaterial that has been identified as promising for the development of amperometric biosensors. Urease, in combination with TRGO, allowed us to create a mediator-free amperometric biosensor with the intention of precise detection of urea in clinical trials. Beyond simplicity of the technology, the biosensor exhibited high sensitivity (2.3 ± 0.1 µA cm^−2^ mM^−1^), great operational and storage stabilities (up to seven months), and appropriate reproducibility (relative standard deviation (RSD) about 2%). The analytical recovery of the TRGO-based biosensor in urine of 101 ÷ 104% with RSD of 1.2 ÷ 1.7% and in blood of 92.7 ÷ 96.4%, RSD of 1.0 ÷ 2.5%, confirmed that the biosensor is acceptable and reliable. These properties allowed us to apply the biosensor in the monitoring of urea levels in samples of urine, blood, and spent dialysate collected during hemodialysis. Accuracy of the biosensor was validated by good correlation (R = 0.9898 and R = 0.9982) for dialysate and blood, utilizing approved methods. The advantages of the proposed biosensing technology could benefit the development of point-of-care and non-invasive medical instruments.

## 1. Introduction

According to a review on biosensing technologies, the use of biosensors for medical technologies has increased exponentially [1]. Since urea level and fluctuation is one of the most important indicators of possible kidney and liver dysfunction, earliest possible detection and real-time monitoring of this metabolite in human body fluids, such as urine or blood, occupies a significant place in clinical biochemistry [2,3,4]. It is known that increased plasma/serum urea concentration can cause heart failure, dehydration, hypovolemic shock, gastrointestinal bleed, and catabolic state (trauma, severe infection) [3]. Moreover, abnormal urea levels may also indicate urea cycle disorders [3,5]. According to a recent report, blood urea nitrogen (BUN) is the most valuable independent risk factor to predict severe acute pancreatitis (AP) [6].

Currently, BUN tests in medical diagnostics have become the most commonly used method to evaluate blood urea levels. However, the main disadvantages of BUN tests are blood sampling, which poses an inconvenience to patients, and subsequent time-consuming logistic channels directed to centralised biochemical laboratories. In general, the use of alternative methods examining urea in body fluids other than blood have great potential [4]. With regard to non-invasive technologies, we started with the creation of an amperometric urea biosensor operating in dialysate. Since this fluid is easily accessible during hemodialysis (HD), electrochemical approaches open up great opportunities for the creation of cost-effective methods. The monitoring of patient urea in real-time can significantly modify HD time by keeping the adequacy of the procedure. The proposed biosensor can also operate in urine, blood, or serum, which further widens the biosensor’s general applicability. Normal urea concentration in blood is 2.5–7.8 mM [3], while the blood of patients suffering from acute kidney injury or chronic kidney disease typically display elevated concentrations—up to 64.3–171.4 mM [7].

Thus far, existing urea biosensors are well presented in several contemporary accounts [2,7,8]. However, most of them are time-consuming, invasive, and utilise non cost-effective technologies, and are, therefore, not suitable for routine monitoring. The possibility to detect urea from the saliva of chronic kidney disease patients using an optical fiber urea sensing system was recently shown [9]. However, this method is rather inconvenient because each measurement requires the addition of a native urease solution.

The majority of urea biosensors are potentiometric analytical systems based on monitoring of pH variation using pH electrodes; however, urease-based potentiometric methods have several disadvantages, such as slower response time and stronger susceptibility to interferences from sodium and potassium ions. Several amperometric urea biosensors presented in reports [8,10] were based on the conversion of compounds possessing an electrochemical activity subjective to pH; this was applied for creation of the urea electrode [11]. However, for the application of such an electrode, an additional reagent-hydrazine has to be applied. Similar urea biosensors were created based on methylene blue, hematein, and lauryl gallate [12]. Sometimes, an additional enzymatic system is used for enhanced sensitivity, which actually rather complicates the exploitation of biosensors [13]. A number of redox polymers were also used as pH-sensitive compounds in urea biosensors [7,12,14,15]. The mechanism of such an action is quite simple. Hydrolysis of urea at pH ca 7 or higher leads to the increased pH and consequently better binding of liberated protons produced during anodic oxidation of the electrochemically active groups in polymers on the surface of carbon electrodes. Anodic current signals correlate with urea concentration [16]. However, the sensitivity of these biosensors strongly depends on the buffer capacity of the system in use. It is worth noting that in many cases, mentioned above, the electrochemical oxidation of carbamic acid acting as an intermediate in the urea decomposition process starts at high potential ca. 1 V vs. Ag/AgCl [13,17]. Such high value of working electrode potential depends on the selected electrode material and is usually a cause of decreased accuracy in measurement due to electro-oxidation of interfering compounds. Despite a large number of developed biosensors, almost all of them have one common disadvantage—a rather narrow linear range of urea determination, counteracted via the use of low-affinity recombinant urease [18]. While many urea sensors have complex layered constructions and rather short storage stability [8], a big challenge still remains in the commercialisation of these technologies. Considering that urea sensors have the potential to become a billion-dollar market [8], it is possible to meet the demand by creating sensors possessing the following characteristics: cost-effective construction, simple and clear mechanism of action, widest latitudes of measuring range, beneficial storage stability, and high sensitivity of response not influenced by interfering compounds.

Graphene-based nanomaterials have been widely investigated for the development of enzyme-based amperometric biosensors due to their electrical conductivity, high surface area, low prices, and good biocompatibility compared to other raw materials [19,20,21]. The large surface is beneficial for the immobilisation of enzymes, while its conductivity, small band gap, and electroactive functional groups have a positive effect for the transfer of electrons between the enzyme and the carbonaceous nanomaterial surface [22]. Thermally reduced graphene oxide (TRGO) has important properties—the presence of electroactive oxygen containing functional groups such as quinones, which can take part in electron transfer (ET) with redox proteins [23,24]. Due to oxygen functional groups, the TRGO possesses the ability to transfer/receive electrons directly to/from enzymes or electroactivate their products, bypassing the need for an additional ET mediator. For biosensors, the absence of mediators is the main advantage, providing them with superior selectivity; both because they operate in a potential window closer to the redox potential of the enzyme itself, and therefore, are less prone to interfering reactions, and also because of the lack of yet another reagent in the reaction sequence [23,25]. Therefore, this carbonaceous nanomaterial could be very promising for amperometric urea biosensor creation.

In this work, we discuss the amperometric biosensor technology using enzyme urease working in synergy with TRGO. Since the proposed biosensor operates without any additional electron transferring compounds, it demonstrates beneficial advantages for the creation of new cost-effective diagnostic tools.

**Figure 1:**
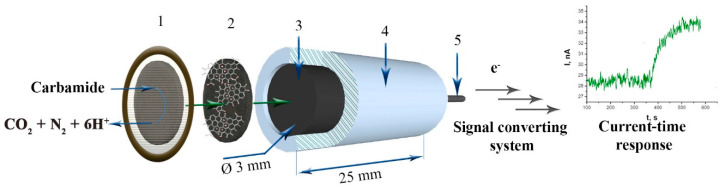
Principle scheme of the thermally reduced graphene oxide -based urea biosensor. 1 and 2—adjustable membrane containing semipermeable terylene film (1) and TRGO layer with immobilized urease (2), 3—electrode contact zone, 4—housing, 5—connection to a response registration device.

The urease (EC 3.5.1.5) was immobilised into a synthesized TRGO layer by forming an adjustable membrane as a separate element, which can easily be replaced when the biosensor sensitivity decreases (Figure 1). From a practical point of view, such TRGO-urease based biosensor design, besides other advantages, demonstrates great physical stability and has been proven to be suitable to work at a potential near 0 mV vs. Ag/AgCl that additionally boasts reduced electrochemical interference found in real samples such as urine, blood, or spent dialysate. Such a system allowed us to detect urea in urine, blood, and spent dialysate with heightened accuracy in a wide range of concentrations, with good reproducibility, and good operational and storage stability. The ET mediator-free action mechanism of the TRGO-based urea biosensor was studied.

## 2. Materials and Methods

### 2.1. Materials

The graphite rod (diameter 3 mm, length 150 mm, Sigma–Aldrich, Germany); urease from *Canavalia ensiformis* EC. 3.5.1.5 (specific activity 1378.0 U mg^−1^, Sigma, USA; bovine serum albumin ((BSA) Merck KGaA, Germany); semi-permeable terylene membrane (thickness 12 µm, pore diameter 0.4 µm, Joint Institute of Nuclear Research, Russia); graphite (extra pure grade Merck, Germany); 1,6-Hexamethylene diisocyanate ((HMDI) Fluka, Switzerland), poly(vinyl alcohol 100,000 ((PVA) Fluka, Switzerland) degree of hydrolyzation 86–89 mol%. Other chemical reagents were obtained from Sigma–Aldrich and were of analytical grade, unless otherwise mentioned. As default buffer, 20 mM sodium phosphate buffer solution ((PBS) Sigma–Aldrich, USA) containing 100 mM of KCl, pH = 7.2 was used. A stock solution was created of urea 1 M in PBS.

The second fraction of TRGO was synthesized from the natural graphite according to the protocol reported by Šakinytė et al. [23]. In this work, thermal reduction and fractionation equipment was used to reduce graphene oxide, which was initially synthesized by the modified Hummer’s method. First, dry graphene oxide (GO) powder crushed in an agate mortar was added to the separator funnel and sealed. The furnace was heated to 800 °C and GO powder from the sealed separator funnel was subjected to the hot zone in small portions (~0.05 g). During this part of the experiment, Ar flow rate was maintained at 100.0 mL min^−1^. Thermally reduced GO particles are moved away from the hot reaction zone by the Ar flow, settling in subsequent chambers. The collected fractions of reduced GO are labeled as TRGO1, TRGO2, and TRGO3, respectively [23].

### 2.2. Preparation of Inert and Enzymatic Membranes

On the surface, a semi-permeable terylene film was fixed to a rubber ring (inner diameter of 3 mm). 0.1 g TRGO powder was stirred at 25 °C for 30 min with the 500 µL pasting liquid consisting of 10% polyvinyl dichloride in acetone; the urease/albumin mixture was immobilized on polyurethane particles (polyurethane particles from PVA and HMDI were synthesized by one-step method in dimethyl sulfoxide/water, then immobilization of 1540 U urease was carried out [26,27]). Aiming to design enzymatic membranes, 100 µL of the prepared mixture was deposited on the inner surface of the ring-fixed membrane and then left at 4 °C overnight.

Inert membranes were prepared in the same manner; only urease was replaced with a corresponding amount of albumin.

### 2.3. Preparation of Electrodes and Biosensor

Aiming to design working electrodes, graphite rods (electrode surface area of 0.07 cm^2^) were polished, glued in Plexiglas tube (length of 25 mm), and covered with an inert terylene membrane. The biosensor was designed by mechanically attaching a membrane containing immobilised urease, instead of the inert membrane, to the surface of the graphite electrode and thoroughly washed with PBS.

### 2.4. Electrochemical Measurements; Determination of Urea in Urine, Blood, and Dialysate

The electrochemical cell (1.65 mL) was supplied with a platinum auxiliary electrode, saturated Ag/AgCl reference electrode, and working electrode (with inert or enzymatic membrane). The electrochemical cell was filled with 1.65 mL of PBS; afterwards, in a case of biosensor calibration, the samples of 20; 25; 30; 35; 40; 45; 50; 60; 70; 80; 90 and 100 µL containing urea in PBS were added. For blood/serum or urine 10 times diluted with PBS, 50 µL of the samples were added to the cell. For the dialysate analysis, 100 µL of the dialysate was added to the cell. The current-time response of the electrodes at working potential 0.2 V vs. Ag/AgCl was recorded using the Advanced Electrochemical System PARSTAT 2273 (Princeton Appl., Res., USA).

Apparent maximum current density ($j_{\max}^{\mathrm{app}}$) and apparent Michaelis constant ($K_{M}^{\mathrm{app}}$) were evaluated from the experimental dependence of the current density (j) vs. concentration of the substrate (C). For this, the response current density was measured three times in the solution with C and the average response j was obtained. The experimental dependence j vs. C was approximated by OriginPro 8 (free trial version from http://www.originlab.com, OriginLab Corporation, USA) according to the electrochemical version of the Michaelis-Menten Eq. [28]. Sensitivities of the biosensors were defined as a slope of the linear range of calibration curve. The detection limit (LOD) was calculated as the standard deviation of the current of response to the substrate (in the linear calibration curve range) multiplied by three and divided by the sensitivity [29]. The inactivation constants (k_in_) for amperometric responses of the TRGO-based urea biosensor were calculated as a slope, obtained in semi-logarithmic coordinates of the responses vs. time. The standard addition method was applied to calculate analytical recovery of the urea biosensor. The recovery for the TRGO-based urea biosensor was determined by testing four urea concentrations: 0.3 mM, 0.6 mM, 1.2 mM, and 1.8 mM by adding the same volume (50 µL) of whole blood specimens or 10 times diluted urine containing 0.25 mM, 0.5 mM, 1.0 mM, and 1.25 mM of urea.

## 3. Results and Discussion

### 3.1. Principles of the TRGO-Based Urea Biosensor and Summarized TRGO Characteristics

The development of the diagnostic device started with an amperometric biosensor using TRGO as an electrode material and urease as a urea recognition element. The prototype amperometric urea biosensor was constructed by coupling an enzymatic membrane consisting of urease and synthesized carbonaceous nanomaterials with the graphite electrode surface. The biosensor construction is presented in Figure 1.

As can be seen in Figure 1, the biosensor has a rather simple construction that allows it to be incorporated into registered devices, as well as to be miniaturised (aiming to create portable constructions). Meanwhile, immobilised urease catalyses the hydrolysis of urea, which in turn cause the effects on increasing of current-time response of the biosensor. The adjustable membrane containing immobilised enzyme performs the double function of high stability and selectivity, especially when acting in urine, blood, or dialysate.

Key aspects of urea biosensor action depend on properties of the incorporated nanomaterial in the adjustable membrane. One of the most commonly-used chemical methods to prepare the graphene-based nanomaterials with electroactive oxygen functional groups is the chemical oxidation of graphite to graphene oxide (GO) and consequent thermal decomposition in an inert atmosphere. For this reason, aiming to create an effective urea biosensor, three TRGO fractions (TRGO1, TRGO2, and TRGO3) were synthesized from natural graphite according to a new thermal reduction protocol reported by Šakinytė et al. [23]. The structural characteristics and surface morphologies of the TRGO fractions were evaluated using X-ray diffraction (XRD), Raman spectroscopy, Brunauer-Emmett-Teller (BET) measurements, and elemental analysis [23]. The summarized data of the key characteristics of the GO, graphite, and TRGO fractions are presented in Table 1.

It is worth mentioning that biosensors based on GO or pristine graphite either show or do not show minimal response (in the case of graphite) to urea. Pristine graphite is characterized by a certain ordered structure, relatively high conductivity and rather small S_BET_, and small amount of defects (I(D)/I(G) = 0.4). This crystalline structure without any function groups is not favorable for urea biosensors. In contrast, a GO-based biosensor does not show any response to the urea probably due to very high amounts of oxygen functional groups (49.8 w%) and defects (I(D)/I(G) = 1.25), disrupted sp^2^ bonding network and, consequently, lower electrical conductivity [30]. Meanwhile, most properties of TRGO were intermediate between those of GO and graphite (crystalline structure, amount of oxygen functional groups and conductivity). Likewise, particles of TRGO fractions possess highest amount of defects as well as S_BET_. Generally, these boundary properties of TRGO nanomaterials determined effective action of the urea biosensor. Due to the highest S_BET_ and optimal amount of oxygen functional groups compared to TRGO1 and TRGO3, the second fraction of thermally reduced graphene oxide (this fraction is marked TRGO in the text below) was selected for the development of the urea biosensor.

#### 3.1.1. Functioning of the TRGO-Based Electrode

Urease catalyses the hydrolysis of urea by yielding ammonia and carbamic acid, which spontaneously decomposes into carbonic acid and second ammonia molecule [31]. Our previous study [32] showed that a carbon black (CB) and immobilized urease-based biosensor exhibits three different actions, depending on the applied CB electrode potential.

**Figure 2:**
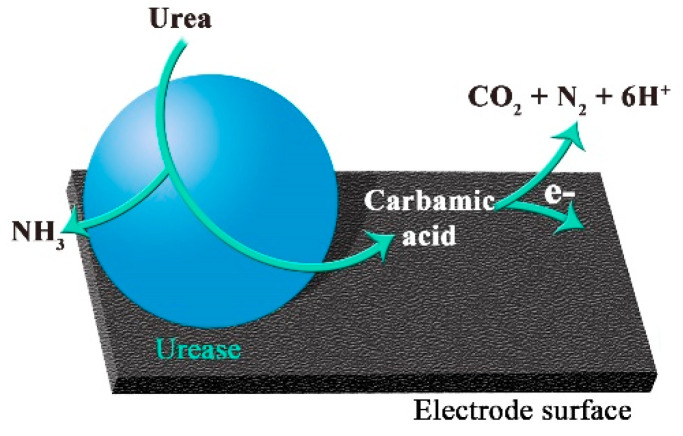
The principle of direct carbamic acid oxidation on carbonaceous nanomaterials’ surface, E = 0.2 V vs. Ag/AgCl.

At low potentials (0–0.1 V vs. Ag/AgCl) of the working electrode, one-electron electrochemical oxidation of carbamic acid can be monitored; meanwhile, at 0.2–0.5 V vs. Ag/AgCl electrode potentials, electro-oxidation of carbamic acid and hydrazine were observed [32]. However, with the TRGO-based biosensor working at applied potential of 0.2 V vs. Ag/AgCl, we assume that in our system, the principle of detection of urea is based on direct carbamic acid oxidation (DCAO). The principle of DCAO on carbonaceous nanomaterials’ surface is presented in Figure 2.

As shown in Figure 2, in presence of urease, urea is hydrolyzed to carbamic acid and ammonia [31]. TRGO has electroactive oxygen containing functional groups such as quinones [23,24], which can take part in ET, with carbamic acid. Thus, carbamic acid electrochemically oxidizes on the electrode surface by generating anodic current, correlating to urea concentration. In this case, oxygen containing functional groups of TRGO participate in electron and proton transfer at the same time. For this reason, electrochemical measurements can be carried out at a low electrode potential of 0.2 V vs. Ag/AgCl. It is worth noting that TRGO works as a transducer and enzyme supporter at the same time, which gains double benefit and in general allows for the creation of various miniaturised biosensor constructions in the simplest form.

Obviously, the urease catalyzed reaction displays feedback that results in pH changes. For detection of such pH changes, we proposed TRGO-based electrodes, since the thermal reduction procedure leads to the formation of specific oxygen groups (quinones, carboxy, lactone, epoxy, phenolic, and carbonyl) that are capable of taking part in proton transfer on the surface of TRGO [24].

**Figure 3:**
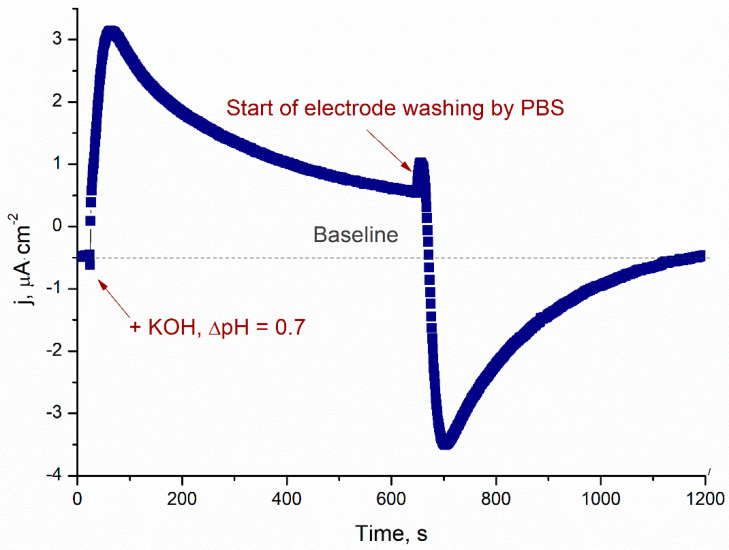
Response of the TRGO-based electrode to an increased pH value of ∆pH 0.7. Rinsing with default PBS (pH 7.2) starting from 620 s. Applied electrode potential 0.2 V vs. Ag/AgCl.

In contrast to TRGO, anodic response to the increased pH on bare graphite electrode is very small because the surface contains only a very small amount of functional oxygen groups. While the TRGO-based electrode surface possesses much more of such derivatives, it determines a higher, but also slightly slower, response. The response becomes longer because the layer of the TRGO particles creates a diffusion limited signal. The TRGO-based electrode can operate at 0 V vs. Ag/AgCl, but an optimal potential of 0.2 V vs. Ag/AgCl for real applications was selected. This decision allowed us to gain the double benefit of urea biosensors—good sensitivity and increased accuracy of analysis. High accuracy of urea monitoring can be reached because at low working potential, only a restricted number of compounds can interfere with the electrochemical response of the biosensor. Although at higher electrode potential the sensitivity of the biosensor can be increased, inexpedient irreversible amination of the electrode matrix also begins. Thus, value of working electrode potential of 0.2 V vs. Ag/AgCl was chosen as optimal. Typical responses of the TRGO-based electrode at 0.2 V vs. Ag/AgCl to the increase of pH value following the addition of a default buffer are presented in Figure 3.

When pH value was increased on 0.7 pH units, anodic current density of the electrode was rapidly raised to 3.1 µA cm^−2^. This means that rapid deprotonation of the TRGO surface active groups and their rapid oxidation did indeed take place. However, oxidation and reorganisation of deeper layers of the TRGO particles is slower, demonstrating the following observation of the electrode response decrease as a first order process (k_in_ = 2.7·10^−3^ s^−1^) (Figure 3). Due to rinsing with default PBS, pH was restored up to 7.2, and active groups on the surface protonates and reduces almost in the same manner as in the oxidation stage. The sensitivity of the proposed pH electrode was found to be 5.1 µA pH^−1^ cm^−2^ (Figure 3), which is roughly 10 times higher than other healthcare pH electrodes [33].

#### 3.1.2. Calibration Characteristics of the TRGO-Based Urea Biosensor

Generally, immobilisation of urease into a layer of TRGO, whilst preparing membranes for urea biosensors does not change the pH response of the electrode; only responses become a bit slower. The thick layer of the TRGO increases the time of relaxation in response, as well as the time of the regeneration of the biosensor. However, an increase in the amount of TRGO particles on the electrode surface can significantly increase the sensitivity of the response as well.

**Figure 4:**
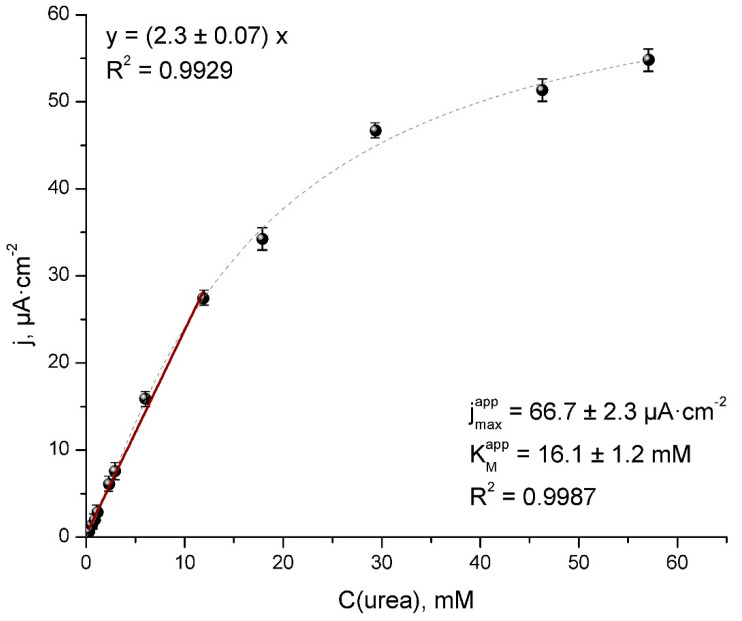
Calibration curve and linear range (black line) for urea obtained using amperometric TRGO-based urea biosensor at 0.2 V vs. Ag/AgCl in 0.02 M of PBS containing 0.1M of KCl.

The TRGO-based urea biosensor response time was about 25 s, which is defined as the time necessary for the current to reach maximum response. The catalytic current as a function of urea concentration is presented in Figure 4.

Figure 4 displays a good linear relationship in the concentration range between 0.2 to 12.0 mM with a sensitivity of 2.3 ± 0.1 μA mM^−1^ cm^−2^. The LOD is considered to be three times higher in noise with a precision of 4% expressed as the relative standard deviation for 10 replicates—calculated as 0.02 mM. The determined values of the sensitivity are higher than the others urea biosensors, based on pH level measurements [14,34] and the same order [35] or slightly lower [10] than urea biosensors, based on an NH_4_^+^ selective electrode. Meanwhile, the linear range is wide enough for blood or spent dialysate samples containing abnormal urea concentration and can be processed without any further treatments.

From the graph, we can also see the main kinetic characteristics of the biosensor: $j_{max}^{app\;}$ = 66.7 ± 2.3 µA cm^−2^, $K_{M}^{app}\;$ = 16.1 ± 1.2 mM. In general, much higher value of $K_{M}^{\mathrm{app}}$ compares to the value of 1.3 mM for native urease acting in solution [36], indicating the presence of a significant diffusion barrier for urea and the reaction layer [28]. From our findings, we can conclude that the majority of structural defects and high surface area of the TRGO [23] leads to a higher values of $K_{M}^{\mathrm{app}}$, which means a higher diffusion barrier and consequently; an extended linear range of the biosensor (Table 1). The diffusion limitations slightly prolonged the response time (up to 25 sec), which was the single disadvantage of the biosensor construction since other TRGO properties, such as the amount of oxygen containing functional groups, positively affected the proper orientation and stability of urease as well as accelerated the ET between the electrode and the enzyme.

The obtained high current density (66.7 ± 2.3 µA cm^−2^) shows that this system can be promising as a bioanode in biofuel cells as well. Moreover, the high sensitivity of the biosensor allows for the application of automatic dilution of real samples in the cell with PBS (34 times for blood and 17.5 times for dialysate). Generally, this procedure stabilises pH fluctuations in different samples, improves metrological parameters, and extends the linear range of the analytical system up to 340 mM of the urea in the sample.

Table 2 provides the parameters of recently (2015–2019 year) reported urea biosensors. According to the table, the proposed TRGO-based urea biosensor has longer storage stability, better sensitivity, and a wider linear range.

As can be seen from Table 2, the determined value of LOD is one of the smallest compared to recently reported studies. The biosensor response time of 25 s compared to other sensors presented in Table 2 is rather slow, but suitable for point-of-care analysis [44] and even faster than 95 among 115, reviewed by Pudrin et al. [8], worldwide presented items.

Considering the good sensitivity and LOD, wide linear range, long storage stability and simple, low-cost construction, the proposed TRGO-based urea biosensor possesses advanced analytical characteristics in comparison with reported studies.

#### 3.1.3. Accuracy of the TRGO-Based Urea Biosensor Acting in Urine, Blood, or Dialysate

The selectivity of urea analysis using the proposed biosensor was determined on two aspects—design of the enzymatic membrane and rather low working potential.

The accuracy of the urea biosensor can be reflected by the recovery test. Analytical recovery for the TRGO-based biosensor was determined by testing four urea concentrations: 0.3 mM, 0.6 mM, 1.2 mM, and 1.8 mM by adding the same volume of whole blood specimens or diluted urine further containing 0.25 mM, 0.5 mM, 1.0 mM, and 1.25 mM of urea. The results including added and found urea concentrations, standard deviation, and recovery are shown in Table 3.

The biosensor demonstrated good recovery. The recovery rate in the range of 92.7–96.4% and 101–104% RSD from 1.0 to 2.5% and from 1.2 to 1.7% was calculated in blood and urine, respectively. This suggests that the TRGO-based urea biosensor exhibits analytical performance possessing high reliability in electrochemical analysis of urea without the interference of other redox-active species in blood or urine samples.

**Figure 5:**
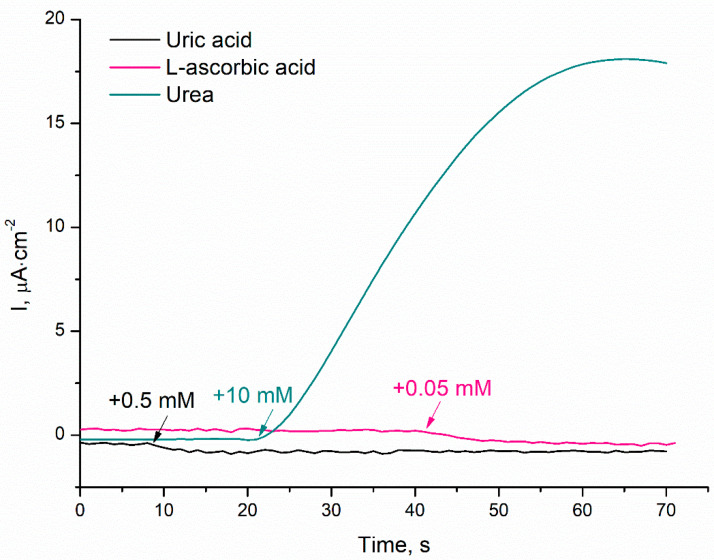
The current-time responses of the TRGO-based urea biosensor on addition of 10 mM of urea, 0.05 mM of L-ascorbic acid, and 0.5 mM of uric acid. Default PBS, applied electrode potential 0.2 V vs. Ag/AgCl.

The amperometric response of the TRGO-based urea biosensor was examined in the presence of common interfering agents, such as L-ascorbic and uric acid. The actually detectable concentrations in urine of 0.05 mM and 0.5 mM of L-ascorbic and uric acid [45] were added into the electrochemical cell and the responses were compared to the response of 10 mM of urea (Figure 5).

Although cathodic current-time responses for L-ascorbic acid and uric acid were slightly visible, these are negligible compared to the signal for 10 mM of urea, as the responses to L-ascorbic acid and uric acid were less—2.5% and 1.2%, respectively.

In the case of spent dialysate, the interference of compounds other than urea was checked with inert membranes, where urease was replaced with a corresponding amount of albumin (see Section 2.2.). No significant detectable responses to samples of dialysate were detected.

Such selectivity towards the target analyte displayed the adaptability to be used for various biologic samples and can be attributed to the low working potential of 0.2 V vs. Ag/AgCl, selectivity of urease enzyme, and specificity of membrane construction.

#### 3.1.4. Operational and Storage Stability of the TRGO-Based Urea Biosensor

The operational stability of the TRGO-based urea biosensor was investigated within 20 days. The responses to 3 mM of urea in PBS were periodically recorded at a working potential of 0.2 V vs. Ag/AgCl. About 30 measurements were done per day. Between the experiments, the biosensor was stored in an electrochemical cell containing PBS. After 20 days, the biosensor sensitivity retained not less than 65% of its initial current response. The RSD of 2% was calculated for 10 urea assays performed over a 2 h period on the fourth measurement day. The biosensor also presented stability for the accomplishment of more than 300 samples of blood or dialysate without significant change. It should be noted that such strong operational stability makes the analysis cost-effective, which is rarely reported.

The storage stability of the TRGO-based biosensor was investigated by intermittent measurements of its response to 3 mM of urea. The obtained biosensor exhibited storage stability for up to seven months with no apparent loss of activity after dry storage at 4 °C. This long-term stability is considerably longer than recently reported urea biosensors (Table 2) and reviewed in [8].

### 3.2. Urea Assessments Using TRGO-Based Biosensor in Spent Dialysate or Blood

Despite absent, well-expressed stationary responses of the amperometric urea biosensor, registration of peak-type signals in urea assessments do not cause any difficulties. The TRGO-based urea biosensor was rather slow, but found to be suitable for point-of-care analysis, and stable and reproducible responses with good sensitivity. These characteristics of the TRGO-urease based biosensor requires very small volume of sensing sample (∼50 μL), making it more suitable for commercialization and is better than present commercial sensors. Thus, we intended to apply the developed system for measurement of urea concentrations in real samples such as blood and dialysate with a consequent comparison of the obtained results with control methods to demonstrate the usability of the biosensor system in medical applications.

**Figure 6:**
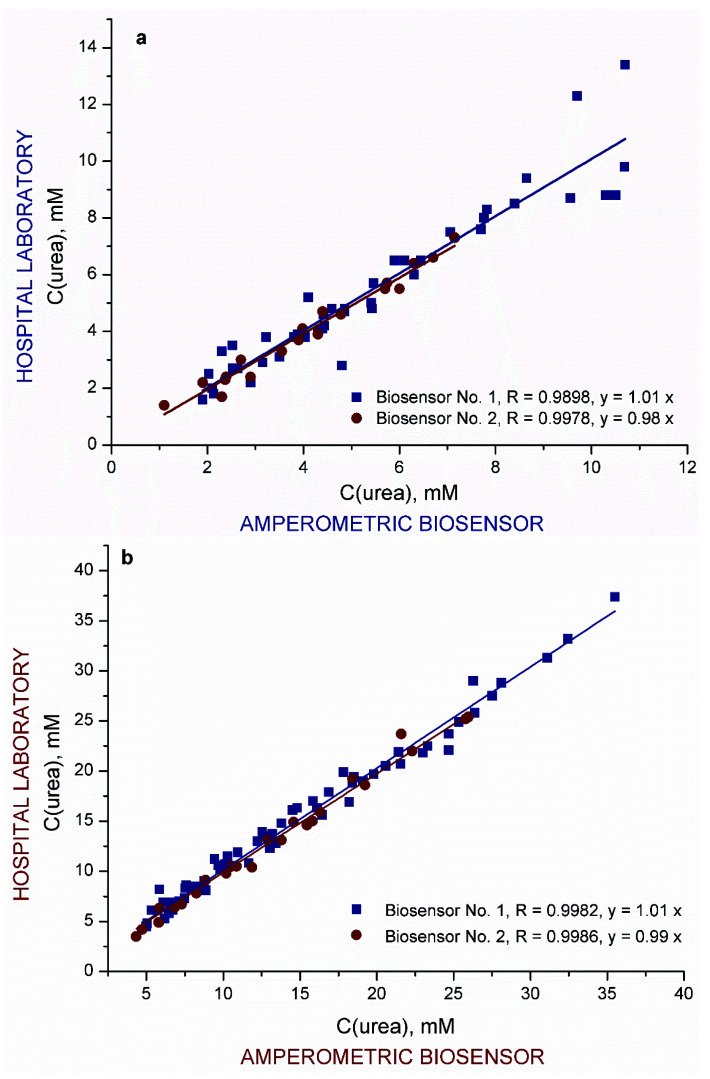
Correlation between urea concentrations obtained in dialysate (**a**) and blood (**b**) using amperometric TRGO-based urea biosensors and in a controlled hospital laboratory.

Aiming to validate responses of the biosensor measurements, the samples of blood and dialysate in parallel were examined at Vilnius University Hospital (VUH) Santariskiu Clinics Laboratory of Biochemistry with the ARCHITECT plus ic8200 analyzer (Abbot Lab., USA). The testing was carried out by investigating the dialysate of 25 cases of HD procedure. The data obtained by both methods are presented in Figure 6a,b. Two TRGO-based biosensors were applied for each urea assessment in dialysate (a) and blood (b).

As can be seen, the correlation of data obtained using the biosensor with hospital laboratory data is good and does not depend on the biosensor applied. Some scattering of the data was observed at high concentrations of urea in the dialysate. High increasing concentrations of urea in dialysate are observed at the beginning of the HD. Samples of the dialysate were collected for the hospital laboratory and the biosensor analyzer into different tubes and could have differed in time by roughly one minute. This experimental delay could cause differences in the urea concentrations in the samples. Observing the correlations (Figure 6), it can be concluded that the proposed TRGO-based biosensor can be effectively used for accurate analysis of samples with different concentrations of urea.

In our work, we started with a urea biosensor operating in spent dialysate, firstly, because it is an approach toward non-invasive technologies and, secondly, because this fluid is easily accessible during an HD procedure. Urea level is currently used as the standard marker for dialysis adequacy and is obtained by calculation of clearance index Kt/V [46]. Nevertheless, Kt/V has several disadvantages: it is complex and tedious to calculate, it ignores the mass transfer between body compartments and across the plasma membrane, which has been shown to be important for the clearance of molecules, practical use of Kt/V requires adjustment for a rebound of the urea concentration due to the multi-compartmental nature of the body, the volume of water per weight of human body needed for Kt/V calculations depends on gender, body size, hydration status, etc [47,48]. Usually the most popular time of in-center HD is calculated theoretically, overlooking these important aspects, and most often, the safe time is selected as four hours [47]. The assessments of 25 cases of HD revealed that urea monitoring in real time can significantly modify the time of HD procedure and could be the best and simple approach to quantify adequacy of HD. Our results obtained in spent dialysate and accordingly in blood using the TRGO-based urea biosensor allowed us to evaluate correlations of urea concentration in blood and in spent dialysate mathematically and also indicated that the duration of procedure could be reduced up to 3 h and in a few cases up to 2 h, instead of the provided 4 h (data not showed) since, up to this time, the patients’ blood possessed a normal level of urea (less than 8 mM). Considering our previous mathematical approach [49], prediction of HD adequacy can be made only by measuring urea in spent dialysate and using an algorithm designed on a base of obtained correlation of urea concentration in blood and spent dialysate. This type of monitoring could be helpfully implemented in adequacy calculation for patients undergoing incremental dialysis, and home-based treatments-determining peritoneal dialysis transport type or choosing a regimen for home HD patients, where HD frequency and duration varies, starting from that similar to in-center dialysis and ending with overnight treatment five times a week [50]. The experiments confirmed that urea measurement in spent dialysate using a TRGO-based urea biosensor possess great potential as a tool for evaluation of dialysis adequacy and also a step leading to point-of-care non-invasive technologies, where mathematical algorithms will replace examination of blood.

## 4. Conclusions

In this work, we proposed an amperometric biosensor based on urease acting in synergy with TRGO. Generally, TRGO nanoparticles used in membrane work as an enzyme supporter and as a response transducer at the same tame. This offer a double benefit and allows the creation of various cost-effective biosensor constructions in the simplest way, this making them suitable for commercialization. The TRGO contains electrochemically active groups that can easily participate in bioelectrochemical reactions at low potential. This phenomenon was applied for the creation of an amperometric pH sensor (sensitivity 5.1 µA pH^−1^ cm^−2^). The sensor possesses a peak-type amperometric response due to a limited number of redox-active groups taking part in electrochemical conversions. Studies on the TRGO-based electrochemical action mechanism revealed that both aspects—catalytic rate of urease and rate of inactivation of the electrochemically active surface—influenced the impact of the pH shift as well as the duality of the biosensor response.

Due to high S_BET_ (689.5 ± 11.3 m^2^ g^−1^) and an optimal amount of oxygen functional groups of TRGO (9.7 w%), the urea hydrolysis product—carbamic acid in the urease catalysed reaction—was electrochemically oxidized on the TRGO surface at low potential. The TRGO-based biosensor showed a high sensitivity of 2.3 ± 0.1 µA cm^−2^ mM^−1^ with a good linear relationship in the concentration range between 0.2 to 12.0 mM (LOD = 0.02 mM).

The biosensor showed good operational and storage stability. After 20 days, the biosensor sensitivity retained not less than 65% of its initial current response. The relative standard deviation of 2% was calculated for 10 urea assays performed over a 2 h period on the fourth measurement day. The biosensor also presented stability for the accomplishment of more than 300 samples of blood or dialysate without significant change. Storage stability was not less than seven months.

The obtained analytical recovery of TRGO-based biosensor in urine of 101 ÷ 104% with RSD of 1.2 ÷ 1.7% and in blood of 92.7 ÷ 96.4%, RSD of 1.0 ÷ 2.5%, indicated that analytical recovery of the proposed TRGO-based biosensor is acceptable and reliable.

High selectivity of the TRGO-based biosensor towards the target analyte, displaying adaptability to be used for various biologic samples, was attributed to several factors: the low working potential of 0.2 V vs. Ag/AgCl, the selectivity of urease, and uniqueness of the biosensor membrane construction. The TRGO-based biosensor was also applied for the monitoring of urea levels in samples of blood and dialysate collected during the HD procedure. Accuracy of the biosensor action in blood and spent dialysate was validated by good correlation (R = 0.9898 and R = 0.9982 for dialysate and blood, respectively) with approved methods. Real-time urea monitoring in dialysate revealed that in more than 56% of analysed HD cases, there is the possibility to reduce the duration of the procedure by up to 1–2 h. These experiments using urine, dialysate, and blood confirmed that urea measurement in spent dialysate using a TRGO-based urea biosensor could be a useful tool not only for evaluation of dialysis adequacy, but also towards point-of-care non-invasive technologies. Further studies are underway to optimise TRGO-based biosensor performance for commercial biomedical applications.

## Figures and Tables

**Table 1:** Structural characteristics of graphite, graphene oxide, and thermally reduced graphene oxide fractions [23].

Carbonaceous Materials	Oxygen, w%	S_BET_^1^, m^2^ g^−1^	I(D)/I(G) ^2^
**Graphite**	0.5	12.8 ± 0.1	0.4
**GO**	49.8	11.1 ± 0.2	1.25
**TRGO1**	10.9	316.8 ± 1.1	1.3
**TRGO2**	9.7	689.5 ± 11.3	1.4
**TRGO3**	9.5	503.0 ± 15.7	1.2

^1^ BET surface area. ^2^ D and G band intensity ration of the Raman spectrum of the carbonaceous materials.

**Table 2:** Characteristics of recently reported urease using biosensors.

Biosensor	Sensitivity, µA mM^−1^ cm^−2^	LR ^a^, mM	RT ^b^, s	LOD, mM	RSD ^c^, %	E ^d^, V	Storage Stability	References
**TRGO/urease**	2.3	0.2–12	25	0.02	2	0.2 vs. Ag/AgCl	Retained 100% in 7 month	This work
**Ferrocene-poly(amidoamine) dendrimers/multi walled carbon nanotubes/urease (Fc-PAMAM (G3)/MWCNT)**	1.085	0.2–1.8	3	0.05	1.95–2.54	0.35 vs. Ag/AgCl	Retained 62% in 3 days	[37]
**Self-assembled polyamidoamine grafted multiwalled carbon nanotube (MWCNT-PAMAM) dendrimers/urease**	6.6 nA mM^−1^	1–20	3	0.4	2.79–3.87	0.45 vs. Ag/AgCl	Retained 83% in 15 days	[38]
**Poly(propylene-co-imidazole)/gold nanoparticles/urease**	-	0.1–30	-	0.036	2.43	0.2 vs. Ag/AgCl	Retained 97% in 75 days	[39]
**Indium tin oxide (ITO) coated Polydiphenylamine (PDPA)/Phosphotungstic acid (PTA)/Graphene (Gra) hybrid nanocomposites modified electrode (ITO/PDPA/PTA/Gra-ME)/urease**	1.085 μA μM^−1^ cm^−2^	0.001–0.013	5	0.0001		0.25 vs. Ag/AgCl	-	[40]
**Graphene/polyaniline (PANI)/urease**	0.85	0.12–12.3	5	0.05	-	−0.2 + 0.4 vs. Ag/AgCl	Retained 81% after 15 days	[16]
**Fe_3_O_4_/MWCNT/PANI/urease**	-	1.0–25.0	˂ 3	0.067	-	0.25 vs. Ag/AgCl	Retained 70% after 60 days	[41]
**Macroporous polypyrrole/urease**	0.0432 mA mM ^−1^	0.5–10.82	5	0.208	4.3	0.3 vs. Ag/AgCl	Retained 93% after month	[42]
**Screen-printed carbon electrode/potassium ferrocyanide/urease/glutamate dehydrogenase/NADH**	-	0.05–40	50	0.012	-	0.20 vs. carbon electrode	Retained 84.2% after 6 months	[43]

^a^ Linear range. ^b^ Respone time. ^c^ Relative standard deviation. ^d^ Working potential.

**Table 3:** Analytical performance of the proposed amperometric TRGO-based urea biosensor in blood and urine samples (n = 3). The standard addition method was applied. The first line represents the initial concentration of urea in blood or in urine. Other lines—difference of urea concentration after addition of urea.

Blood				Urine			
Urea Added, mM	Urea Found, mM	Recovery, %	RSD, %	Urea Added, mM	Urea Found, mM	Recovery, %	RSD, %
**0**	0.20		1.6	**0**	0.49		1.3
**0.3**	0.29	96.4	1.3	**0.25**	0.26	102	1.7
**0.6**	0.57	95.3	1.0	**0.5**	0.52	104	1.4
**1.2**	1.11	92.7	1.7	**1**	1.01	101	1.4
**1.8**	1.69	94.0	2.5	**1.25**	1.26	101	1.2
